# A Fungal Metabolite Asperparaline A Strongly and Selectively Blocks Insect Nicotinic Acetylcholine Receptors: The First Report on the Mode of Action

**DOI:** 10.1371/journal.pone.0018354

**Published:** 2011-04-01

**Authors:** Koichi Hirata, Saori Kataoka, Shogo Furutani, Hideo Hayashi, Kazuhiko Matsuda

**Affiliations:** 1 Department of Applied Biological Chemistry, Faculty of Agriculture, Kinki University, Nakamachi, Nara, Japan; 2 Graduate School of Life and Environmental Sciences, Osaka Prefecture University, Nakaku, Sakai, Osaka, Japan; Yale School of Medicine, United States of America

## Abstract

Asperparalines produced by *Aspergillus japonicus* JV-23 induce paralysis in silkworm (*Bombyx mori*) larvae, but the target underlying insect toxicity remains unknown. In the present study, we have investigated the actions of asperparaline A on ligand-gated ion channels expressed in cultured larval brain neurons of the silkworm using patch-clamp electrophysiology. Bath-application of asperparaline A (10 µM) had no effect on the membrane current, but when delivered for 1 min prior to co-application with 10 µM acetylcholine (ACh), it blocked completely the ACh-induced current that was sensitive to mecamylamine, a nicotinic acetylcholine receptor (nAChR)-selective antaogonist. In contrast, 10 µM asperparaline A was ineffective on the γ-aminobutyric acid- and L-glutamate-induced responses of the *Bombyx* larval neurons. The fungal alkaloid showed no-use dependency in blocking the ACh-induced response with distinct affinity for the peak and slowly-desensitizing current amplitudes of the response to 10 µM ACh in terms of IC_50_ values of 20.2 and 39.6 nM, respectively. Asperparaline A (100 nM) reduced the maximum neuron response to ACh with a minimal shift in EC_50_, suggesting that the alkaloid is non-competitive with ACh. In contrast to showing marked blocking action on the insect nAChRs, it exhibited only a weak blocking action on chicken α3β4, α4β2 and α7 nAChRs expressed in *Xenopus laevis* oocytes, suggesting a high selectivity for insect over certain vertebrate nAChRs.

## Introduction

**Figure 1:**
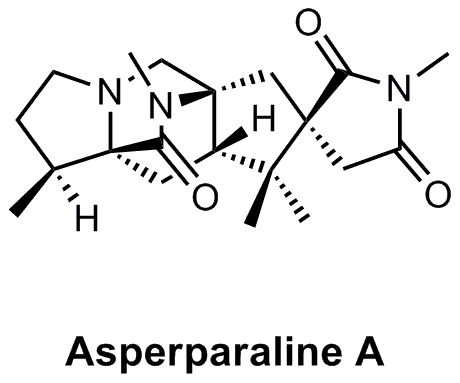
Chemical structure of asperparaline A.

Asperparalines are alkaloids produced by *Aspergillus japonicus* JV-23 when grown on “okara” media (soybean residue resulting from tofu manufacturing). They are known to paralyze silkworm (*Bombyx mori*) larvae when administered orally using artificial diets [1]. Asperparalines A, B and C possess unique 3-spiro-succinimide and cyclopent[f]indolizine moieties along with a *N*-methylamide bridge [2] (Fig. 1). The unique structures of asperparalines have prompted challenges for total synthesis [3], but their targets and selectivity have not yet been elucidated.

It is presumed that the likely target of asperparaline A is the nervous system or neuromuscular junction, since the compound induces paralysis in the silkworm larvae. By applying whole-cell patch-clamp electrophysiology to larval neurons of *B. mori*, we were able to record the neurotransmitter-evoked responses of native ligand-gated ion channels and study the actions of asperparaline A. Having detected a blocking action on nicotinic acetylcholine receptors (nAChRs), we also investigated the actions of asperparaline A on vertebrate (avian) α3β4, α4β2 and α7 nAChRs expressed in *Xenopus laevis* oocytes using two-electrode voltage-clamp electrophysiology. We found that the fungal metabolite specifically and non-competitively blocked the ACh-induced response of the native nAChRs in the insect neurons, but hardly affected receptors for γ-aminobutyric acid (GABA) and L-glutamate. Much weaker blocking actions of asperparaline A were observed on 3 classes (α3β4, α4β2 and α7) of vertebrate (avian) nAChRs, suggesting selectivity for invertebrate nAChRs.

## Materials and Methods

### Approval of this study and animal treatment

This study using living modified organisms (LMO) has been approved by the committee of Kinki University for the experiments involving the production of LMOs (ID number: KDAS-16-015). We used an anesthetic tricaine to reduce the pain of female frogs (*Xenopus laevis*) as much as possible when we removed oocytes from the frogs by referring to the U.K. Animals (Scientific Procedures) Act, 1986.

### *B. mori* neurons

Heads were dissected from last instar larvae of *B. mori* and placed in a Ca^2+^-free physiological saline solution of the following composition: 135 mM NaCl, 3 mM KCl, 4 mM MgCl_2_, 10 mM glucose and 10 mM HEPES (pH 7.3, adjusted with NaOH), supplemented with 50 units ml^−1^ penicillin and 50 µg ml^−1^ streptomycin. The brains were isolated and desheathed using fine forceps and then treated with 1.0 mg ml^−1^ collagenase (Type IA, Sigma-Aldrich Japan, Tokyo, Japan) dissolved in the Ca^2+^-free saline for 30–40 min at room temperature. After washing with the Ca^2+^-free saline, the brains were transferred to a Ca^2+^-supplemented incubation saline of the following composition: 135 mM NaCl, 3 mM KCl, 4 mM MgCl_2_, 5 mM CaCl_2_, 10 mM glucose, 10 mM trehalose and 10 mM HEPES (pH 7.3, adjusted with NaOH) supplemented with 10% fetal bovine serum and 50 units ml^−1^ penicillin and 50 µg ml^−1^ streptomycin. The neurons were dissociated by gentle pipetting using a 1,000 µl micropipette tip, and the resultant cell suspension was placed onto poly-D-lysine (Sigma-Aldrich Japan, Tokyo, Japan)-coated coverslips which were placed in a 35-mm diameter culture dish and left for 60 min. The *B. mori* neurons were then incubated at 25°C for 18–36 h before electrophysiology. All salines used in the cell culture were filter sterilized.

### Whole-cell patch-clamp electrophysiology

The whole-cell patch-clamp electrophysiology [4] was conducted at 20–23°C. The recording electrodes (patch pipette) were prepared from glass capillaries (PG150T-10, Harvard Apparatus, Holliston, MA, USA) using a PE-83 puller (Narishige, Tokyo, Japan). The patch pipette was filled with an internal solution (100 mM KCl, 1 mM CaCl_2_, 4 mM MgCl_2_, 20 mM sodium pyruvate, 10 mM EGTA and 10 mM HEPES (pH 7.3, adjusted with Tris)). Only pipettes having a resistance of 5–6 MΩ when filled with the internal solution were used for experiments. Coverslips with neurons attached were carefully transferred to the recording chamber (RC-16, Warner Instruments, Hamden, CT, USA) and superfused continuously at 5 ml min^−1^ with a physiological saline (135 mM NaCl, 3 mM KCl, 5 mM CaCl_2_, 4 mM MgCl_2_, 10 mM glucose and 10 mM HEPES (pH 7.3, adjusted with NaOH)). The membrane currents were recorded using an Axopatch 200B amplifier (Molecular Devices, Sunnyvale, CA, USA) and low-pass filtered at 10 kHz using a four pole-Bessel filter. Data were stored on a personal computer, for subsequent analysis, using a Digidata 1320A data acquisition system (Molecular Devices, Sunnyvale, CA, USA). The holding membrane potential of the neuronal membrane was −60 mV. The current-clamp method that keeps the membrane current at zero was also used to examine the effect of asperparaline A on the resting membrane potential of the neuron. ACh, L-glutamate and GABA were applied to the *B. mori* neurons using a U-tube; fipronil, mecamylamine and asperparaline A were applied by either U-tube or bath-application.

### Expression of vertebrate nicotinic acetylcholine receptors in *X. laevis* oocytes

Oocytes at stage V or VI of development were removed from female *X. laevis* under anesthetic in 1.5 g l^−1^ tricaine [5], [6], [7]. Oocytes were then treated for 30–40 min at room temperature with 2.0 mg ml^−1^ collagenase (Type IA, Sigma-Aldrich Japan, Tokyo, Japan) dissolved in the Ca^2+^-free standard oocyte saline (SOS) of the following composition: 100 mM NaCl, 2 mM KCl, 1.8 mM CaCl_2_, 1 mM MgCl_2_ and 5 mM HEPES 5.0 (pH 7.6). After washing in Ca^2+^-free SOS to remove collagenase, the follicle cell layer was manually removed using forceps, and followed with the nuclear injection of 20 nl cDNAs of the chicken nAChR subunits (α3, α4, α7, β2 and β3) in the pcDNA3.1 (+) expression vector in distilled water (final concentration of each cDNA: 0.1 ng nl^−1^). For α3β4 and α4β2, 1∶1 mixtures of the α and the non-α (β2 and β3) cDNA solution were injected into oocytes. The injected oocytes were incubated at 18°C in SOS supplemented with penicillin (100 units ml^−1^), streptomycin (100 µg ml^−1^), gentamycin (20 µg ml^−1^) and 2.5 mM sodium pyruvate. Electrophysiology was conducted 3–5 days after nuclear injection of cDNAs.

### Two-electrode voltage-clamp (TEVC) electrophysiology

TEVC electrophysiology was performed at room temperature (18–23°C). The *X. laevis* oocytes were secured in a Perspex recording chamber that was continuously perfused with SOS (7–10 ml min^−1^) as previously described [7], [8]. Membrane currents were recorded using a GENECLAMP 500B amplifier (Molecular Devices, Sunnyvale, CA, USA) at a holding potential of −100 mV. The electrodes were filled with 2 M KCl and had a resistance of 1–5 MΩ when measured in SOS. Signals were digitized using a Digidata 1200 data acquisition system (Molecular Devices) and recorded using Clampex 9.0 (Molecular Devices). Agonists were dissolved in SOS and were applied to oocytes for 3–5 s, with an interval of 1–5 min between applications, to ensure a full recovery from desensitization. Asperparaline A (10 µM) was bath-applied to oocytes for 1 min and then co-applied with ACh.

### Analysis of electrophysiological data

The membrane current data were analyzed using Clampfit 9.2 (Molecular Devices, Sunnyvale, CA, USA). The concentration-inhibition curves for asperparaline A were fitted with the following equation, using Prism 4.03 (GraphPad Software, CA, USA):
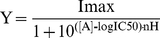
(1)where Y is the normalized response, I_max_ is the normalized maximum response, IC_50_ (M) is the half maximal inhibitory concentration, [A] is the logarithm of the concentration of asperparaline A (M) and n_H_ is the Hill coefficient. On the other hand, the concentration-response curves for ACh were fitted with the following equation:
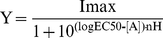
(2)where EC_50_ (M) is the half maximal effective concentration.

### Chemicals

Fipronil and mecamylamine hydrochloride were purchased from Sigma-Aldrich Japan (Tokyo, Japan). Asperparaline A was obtained by purifying the okara broth of *A. japonicus* JV-23 as previously reported [1], [2]. Stock solutions of fipronil, mecamylamine and asperparaline A were prepared in DMSO at a concentration of 10–100 mM and stored at −20°C until use. These stock solutions were diluted with the physiological saline described below. The final concentration (v/v) of DMSO in test solutions was 0.1% or lower, which had no adverse effect on the cellular response under investigation. Test solutions of ACh, L-glutamate and GABA were prepared by directly dissolving the stock solutions in saline immediately prior to experiments.

## Results

### Membrane currents induced by three neurotransmitters in *B. mori* larval brain neurons and actions of asperparaline A on the membrane currents

**Figure 2:**
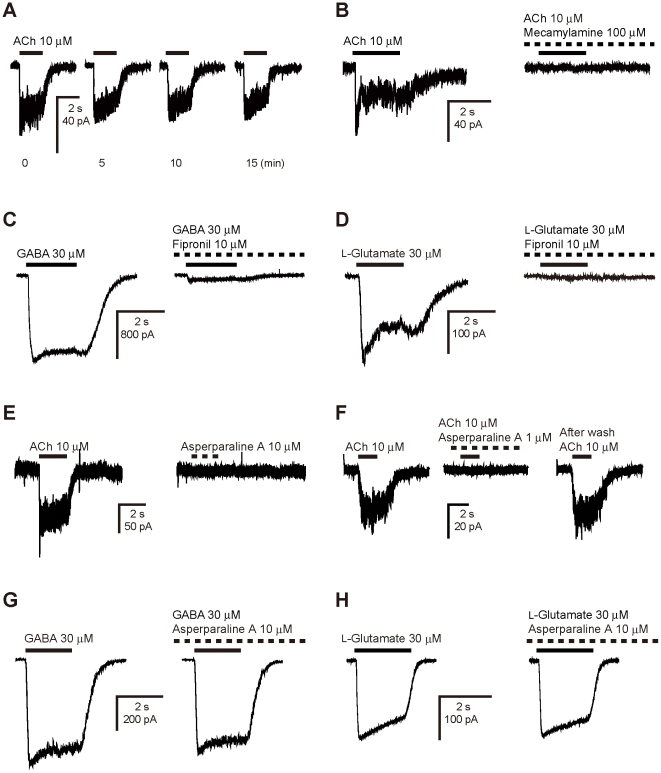
Acetylcholine (ACh)-induced currents (A), the effects of blockers (mecamylamine and fipronil) on the ACh- (B), γ-aminobutyric acid (GABA) (C)- and L-glutamate (D)-induced currents and the actions of asperparaline A on the resting-state (E) and neurotransmitter-evoked currents (F–H) in the silkworm (*Bombyx mori*) larval neurons. The holding potential was −60 mV. ACh (10 µM), L-glutamate (30 µM) and GABA (30 µM) was applied for 2 s using the U-tube, whereas mecamylamine and fipronil were bath-applied for 1 min prior to co-application with the agonists. In (E), asperparaline A was applied alone at 1 µM for 2 s using the U-tube, whereas in (F–H), it was bath-applied for 1 min prior to co-application with neurotransmitters ACh (F), GABA (G) and L-glutamate (H). Note that both peak and slowly desensitizing current amplitudes of the ACh-evoked response were blocked reversibly, selectively and almost completely by 1 µM asperparaline A (F).

Application of ACh (10 µM) resulted in a rapid inward current at a holding potential of −60 mV with fast and slow desensitizing phases. The ACh-induced currents were stably recorded using intracellular (pipette) and extracellular (bath) solutions for 15 min or longer (Fig. 2A). The entire current was completely blocked by bath-applied 100 µM mecamylamine (n = 4, Fig. 2B), a non-competitive antagonist of nAChRs. Both GABA- and L-glutamate-induced currents at the same holding potential were attenuated by bath-applied 10 µM fipronil, a phenylpyrazole insecticide known to block the chloride channels of GABA- and L-glutamate-gated chloride channels in insects (Fig. 2C (n = 4), D (n = 4)) [9], [10].

To examine if asperparaline A activates any of ligand-gated ion channels expressed in the silkworm neurons, it was applied alone to the neurons at 10 µM. Asperparaline A had no effect on the membrane current amplitude to clamp the membrane potential of the *B. mori* larval neurons at −60 mV (n = 4, Fig. 2E). In addition, the compound was also ineffective on the resting membrane potential of the neuron when tested under the current clamp condition (n = 5, data not shown). Hence, it was bath-applied for 1 min, prior to co-application for 2 s with ACh (10 µM), GABA (30 µM) and L-glutamate (30 µM) (These neurotransmitter concentrations are close to EC_50_), to explore any possible antagonist actions on any ligand-gated ion channels present on the neurons. Asperparaline A markedly and reversibly blocked the ACh-induced current when applied at 1 µM (Fig. 2F). However, the alkaloid barely affected the peak current amplitude of the GABA (n = 5, Fig. 2G)- and L-glutamate (n = 5, Fig. 2H)-evoked responses.

### Effects of repeated application of ACh and pre-application on the blocking action of asperparaline A

**Figure 3 pone-0018354-g003:**
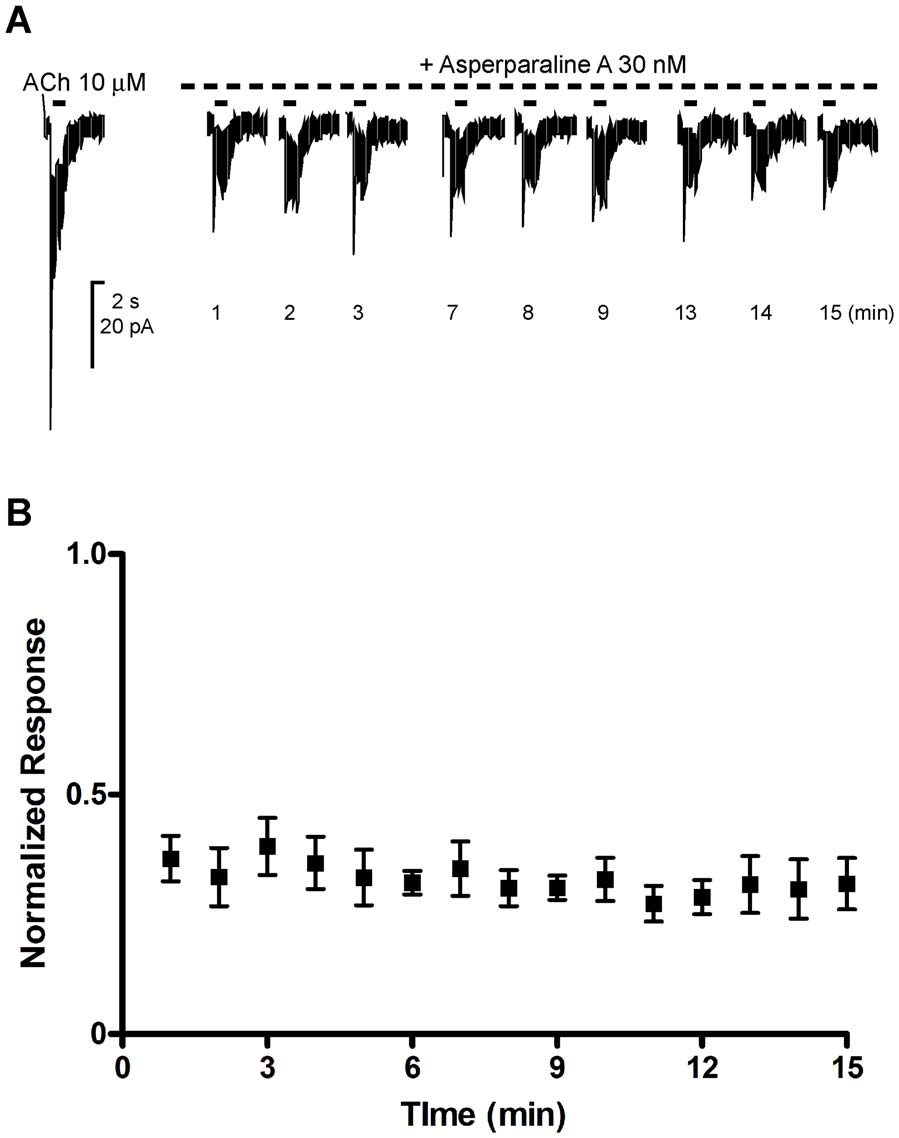
The effects of repeated application of ACh on the blocking action of asperparaline A. After recording the control response to ACh at 10 µM, asperparaline A was continuously bath-applied at 30 nM, during which ACh was also applied at 10 µM for 2 s every minute using the U-tube. (A) Traces of the ACh-induced current responses in the presence of 30 nM asperparaline A. (B) Normalized peak current amplitude of the ACh responses recorded during the continuous application of asperparaline A. The peak current amplitude of each response was normalized by that of the response recorded before the application of asperparaline A. Each plot represents the mean ± standard error of the mean of 4 separate experiments.

To examine whether the blocking action of asperparaline A was use-dependent, asperparaline A was continuously bath-applied at 30 nM, during which ACh was also applied at 10 µM for 2 s every minute. In such experiments, the blocking action was not accelerated by repeated ACh-application over a 10 min period (n = 4, Fig. 3A, B).

**Figure 4 pone-0018354-g004:**
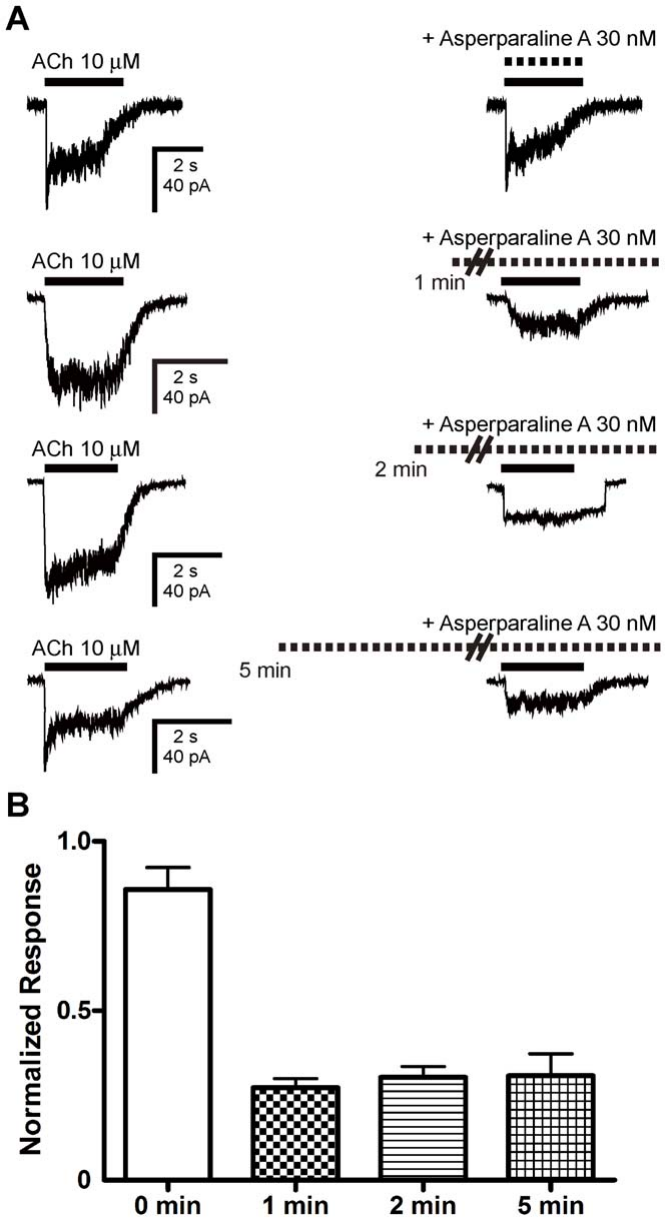
Effects of pre-application on the antagonist action of asperparaline A. (A) Asperparaline A was co-applied at 30 nM with 10 µM ACh for 2 s without pre-application, or applied for 1, 2 and 5 min prior to co-application with 10 µM ACh. (B) The antagonist action of asperparaline A with and without pre-application for 1, 2 and 5 min. Each bar graph represents the mean ± standard error of the mean (n = 4) of the peak current amplitude of the ACh-induced response normalized by that taken before the application of asperparaline A. The pre-application of asperparaline A significantly enhanced the antagonist action (*p*<0.05, One-way ANOVA, Tukey's test), but there were no significant differences in the blocking action between 1, 2, and 5 min pre-applications.

The antagonist potency of asperparaline A observed without pre-application was significantly lower than when pre-applied (n = 4, *p*<0.05, one-way ANOVA, Tukey's test, Fig. 4A, B). Thus, the effects of three different pre-application times (1, 2 and 5 min) on the blocking action were examined. No significant difference in the blocking action was observed between the pre-application times tested (n = 4, Fig. 4A, B).

### Mode of blocking action of asperparaline A on *B. mori* nicotinic acetylcholine receptors

**Figure 5:**
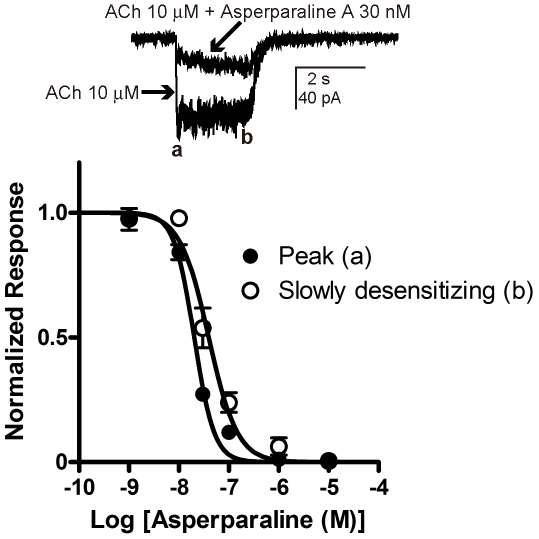
Concentration-inhibition curves for asperparaline A in terms of attenuation of the responses to ACh of the silkworm larval neurons. (A) The ACh-induced responses recorded before and after bath-application of asperparaline A for 1 min prior to co-application with 10 µM ACh. The peak and slowly desensitizing currents are indicated by “a” and “b”, respectively. (B) Concentration-inhibition curves for asperparaline A. Data were normalized to the maximum response to ACh (10 µM). Each plot represents the mean ± the standard error of the mean of 4 experiments. The concentration-inhibition curves were obtained by fitting the data to Eq. (1) (see Materials and Methods). The pIC_50_ ( = log(1/IC_50_) values for the peak and slowly desensitizing currents were 7.69±0.02 (n = 4, IC_50_ = 20.2 nM) and 7.40±0.04 (n = 4, IC_50_ = 39.6 nM), respectively. These two values are significantly different (*p*<0.05, *t*-test).

It has been shown that a neonicotinoid insecticide imidacloprid differentially modulated two phases (desensitizing and non-desensitizing) of the ACh-induced currents in the American cockroach neurons [11]. Hence we examined whether asperparaline A differentially blocks the peak and slowly desensitizing currents. Using the 1 min pre-application protocol, the pIC_50_ ( = log(1/IC_50_) of asperparaline A for the peak and slowly desensitizing current amplitudes were determined to be 7.69±0.02 (n = 4, IC_50_ = 20.2 nM) and 7.40±0.04 (n = 4, IC_50_ = 39.6 nM), respectively (Fig. 5). A significant difference was observed between the two IC_50_ values ((*p*<0.05, *t*-test).

**Figure 6:**
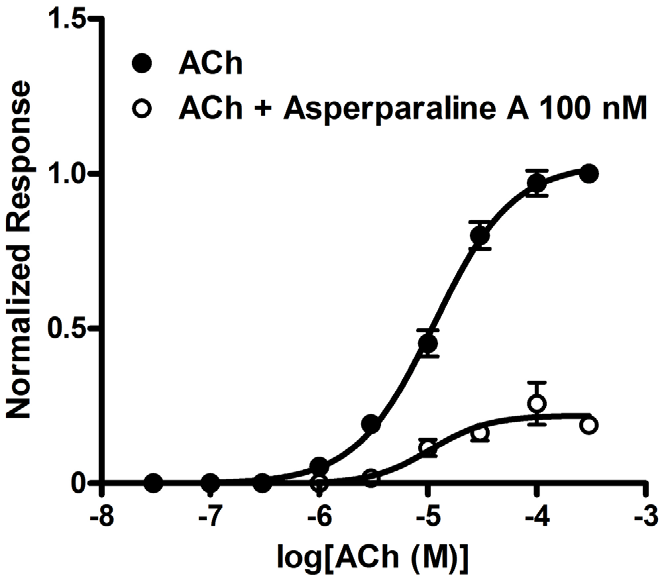
Effects of asperparaline A on the concentration-response curve for ACh in the silkworm larval neurons. The ACh-induced responses were measured at various concentrations in the presence and absence of 100 nM asperparaline A. The concentration-response curves were obtained by fitting the data to Eq. (2) (see Materials and Methods). The pEC_50_ ( = log(1/EC_50_)) values determined in the presence and absence of asperparaline A were 4.98±0.10 (n = 4, EC_50_ = 10.5 µM) and 4.94±0.04 (n = 7, EC_50_ = 11.4 µM), respectively. No significant shift in EC_50_ was observed by the application of asperparaline A.

To explore further the blocking action, the concentration-response relationship of ACh was measured in the presence and absence of 100 nM asperparaline A (Fig. 6) using the 1 min pre-application protocol for the alkaloid application. It reduced the normalized maximum response to ACh to approximately 25.7%, while scarcely influencing pEC_50_ (with 100 nM asperparaline A, 4.98±0.14, *n* = 4, EC_50_ = 10.5 µM; without asperparaline A, 4.94±0.04, *n* = 7, EC_50_ = 11. 4 µM). No significant shift in EC_50_ was observed by the presence of 100 nM asperparaline A.

### Actions of asperparaline A on vertebrate nicotinic acetylcholine receptors expressed in *X. laevis* oocytes

**Figure 7:**
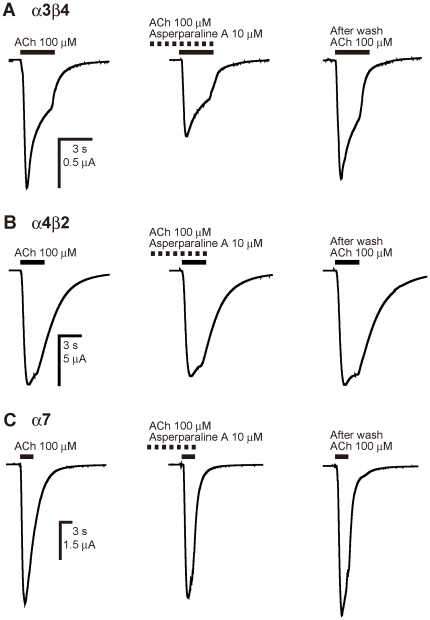
Effects of asperparaline A on the ACh-induced responses of chicken α3β4 (A), α4β2 (B) and α7 (C) nAChRs expressed in *Xenopus laevis* oocytes. After three successive control applications of ACh, 10 µM asperparaline A was continuously bath-applied and then co-applied with 100 µM ACh. Asperparaline A blocked the ACh-response of α3β4 nAChR by 33.4±3.3% (n = 3), whereas it scarcely influenced the response of α4β2 (n = 4) and α7 (n = 3) nAChRs.

Asperparaline A was tested on the chicken α3β4, α4β2 and α7 nAChRs expressed in *X. laevis* oocytes (Fig. 7). When tested alone, the alkaloid showed no agonist action on these three nAChRs, at concentrations up to 10 µM (data not shown). Thus it was bath-applied at 10 µM for 1 min prior to co-application with 100 µM ACh. It reduced the peak current amplitude of the ACh-induced response of α3β4 nAChR by 33.4±3.3% (n = 3, Fig. 7A), while barely influencing the amplitudes of the responses to ACh of the α4β2 (n = 4, Fig. 7B) and α7 (n = 3, Fig. 7C) nAChRs.

## Discussion

Since the discovery of asperparaline A in 1997, its target has remained unknown. Here we have for the first time tested asperparaline A on ligand-gated ion channels present on the silkworm larval neurons using patch-clamp electrophysiology. Asperparaline A was found to selectivity reduce the ACh-induced currents (Fig. 2F) that were also blocked by mecamylamine (Fig. 2B). In addition, it barely affected the GABA (Fig. 2G)- and L-glutamate (Fig. 2H)-induced currents, indicating a specific antagonist action on nAChRs present in the neuron. In insects, however, cation-permeable, ionotropic glutamate receptors mediate fast-acting neuromuscular transmission and are targeted by several venoms [12]. As such, tests of asperparaline A on this type of ligand-gated ion channels are of importance to ensure that the toxicity of this compound to the silkworm larvae is the result of the selective antagonist action on nAChRs.

Asperparaline A was not an open channel blocker of the nAChRs because there was no evidence of use-dependency in the blocking action (Fig. 3). The ACh-induced currents consisted of fast and slow desensitizing phases (Figs. 2–5), which may reflect the presence of several receptor subtypes as reported for other insect neurons [11]. The peak and slowly-desensitizing ACh-induced currents showed different asperparaline-sensitivity (Fig. 5). Given that the isoforms of all the silkworm nAChR subunits resulting from splicing and RNA editing have been elucidated [13], it will be of interest in future to examine the affinity of asperparaline A for nAChR subtypes. Nonetheless, it is at present difficult to express functional and robust nAChRs consisting of only insect receptor subunits including those of the silkworm in heterologous cells, which should be resolved primarily.

We examined the effects of asperparaline A on the concentration-response curve for ACh. The alkaloid (100 nM) reduced the normalized maximum response to ACh, while scarcely influencing EC_50_ (Fig. 6), suggesting that ACh and asperparaline A do not compete for the same binding site at nAChRs.

To investigate whether asperparaline A is a selective antagonist of insect nAChRs, or equally effective on vertebrate nicotinic AChRs, its actions on the chicken α3β4, α4β2 and α7 nAChRs expressed in *X. laevis* oocytes were investigated using two-electrode voltage-clamp electrophysiology. Although α3β4 nAChR showed higher asperparaline A-sensitivity than others, the blocking effect was only 33.4% of the control response at 10 µM, a concentration about 250–500-fold higher than the IC_50_ for the *B. mori* nAChRs (Fig. 7). Moreover, the blocking action on α4β2 and α7 was very weak at this concentration, suggesting a high selectivity for insect over certain vertebrate (avian) nAChRs. We cannot of course rule out that other vertebrate nAChRs may show higher sensitivity to this alkaloid than α3β4, α4β2 and α7 [14].

In conclusion, this is the first study to have shown that asperparaline A from *A. japonicus* JV-23 targets the nAChRs among the ligand-gated ion channels expressed by *B. mori* neurons, offering an explanation, at least in part, for the paralysis exhibited by silkworm larvae exposed to this compound. The asperparaline A acts on native *B. mori* nAChRs as a non-competitive antagonist, and is highly selective to insect (silkworm), over vertebrate (chicken), nAChRs. Future research should focus on elucidation of the mechanism of the selectivity, which may pave a new way for novel pest control chemicals.
